# Tissue Engineering Strategies for Retina Regeneration

**DOI:** 10.3390/app11052154

**Published:** 2021-02-28

**Authors:** Deepthi S. Rajendran Nair, Magdalene J. Seiler, Kahini H. Patel, Vinoy Thomas, Juan Carlos Martinez Camarillo, Mark S. Humayun, Biju B. Thomas

**Affiliations:** 1 Department of Ophthalmology, Keck School of Medicine, University of Southern California, Los Angeles, CA 90033, USA; 2 Departments of Physical Medicine & Rehabilitation, Ophthalmology, Anatomy & Neurobiology, Sue and Bill Gross Stem Cell Research Centre, University of California, Irvine, CA 92697-1705, USA; 3 Department of Physics, University of Alabama at Birmingham, Birmingham, AL 35233, USA; 4 USC Ginsburg Institute for Biomedical Therapeutics, University of Southern California, Los Angeles, CA 90033, USA

**Keywords:** retinal degenerative diseases, age-related macular degeneration, biomaterials, stem cells, retinal pigment epithelium, tissue engineering

## Abstract

The retina is a complex and fragile photosensitive part of the central nervous system which is prone to degenerative diseases leading to permanent vision loss. No proven treatment strategies exist to treat or reverse the degenerative conditions. Recent investigations demonstrate that cell transplantation therapies to replace the dysfunctional retinal pigment epithelial (RPE) cells and or the degenerating photoreceptors (PRs) are viable options to restore vision. Pluripotent stem cells, retinal progenitor cells, and somatic stem cells are the main cell sources used for cell transplantation therapies. The success of retinal transplantation based on cell suspension injection is hindered by limited cell survival and lack of cellular integration. Recent advances in material science helped to develop strategies to grow cells as intact monolayers or as sheets on biomaterial scaffolds for transplantation into the eyes. Such implants are found to be more promising than the bolus injection approach. Tissue engineering techniques are specifically designed to construct biodegradable or non-degradable polymer scaffolds to grow cells as a monolayer and construct implantable grafts. The engineered cell construct along with the extracellular matrix formed, can hold the cells in place to enable easy survival, better integration, and improved visual function. This article reviews the advances in the use of scaffolds for transplantation studies in animal models and their application in current clinical trials.

## 1. Introduction

The human retina, which is situated in the posterior part of the eye is a transparent, light-sensitive tissue containing multiple cellular layers. It originates from the anterior neural tube during early embryogenesis as a part of the central nervous system [1]. The retina is composed of the light transducing neural retina, as well as the supportive blood-retinal barrier. In the neural retina, after absorption of photons of light energy by the photoreceptors (PR)—the rods and cones, the visual information is converted into chemical signals and then to neural signals that are transmitted to retinal ganglion cells (RGC). The RGC axons form the optic nerve that transmits this information to the brain visual centers where the image is processed [2]. The blood–retina barrier consists of a polarized monolayer of hexagonal cells—the retinal pigment epithelial cells (RPE) which support and nourish the PR; Bruch’s membrane (BM)—a specialized basement membrane which transports nutrients to the retina, and retinal vascular endothelial cells of the underlying choroid [3].

Although there are variations in the pathologies typical of retinal degenerative diseases (RDs) including age-related macular degeneration (AMD), retinitis pigmentosa (RP), and Stargardt’s disease (SD), it is currently considered that RPE dysfunction and the resultant deterioration of photoreceptors are the most common pathologies. Furthermore, BM may thicken and alter its composition, resulting in compromised nutrient transport to the retina. Degeneration of RPE and photoreceptors result in significant visual disability which eventually leads to irreversible vision loss. The existing therapies can only delay the progression of retinal diseases, except for anti-angiogenic treatments for patients with neovascular age-related macular degeneration [4]. Currently, there are no established treatment strategies to completely halt the degenerative process or reinstate regular retinal function to restore vision. Although electronic retinal interface devices [5] and gene therapies [6] are under clinical trials, the extent of achievable results is likely a long way from permanent visual recovery.

In many instances of retinal degeneration, even after RPE and PR loss, the inner layers of the retina with its intricate neural connectivity maintain their architecture for an extended period. If a population of healthy RPE/PR are delivered to the subretinal space, they can survive and integrate with the host retina to restore vision. Based on this, a cell replacement strategy is a promising approach for the treatment of AMD and RP. Reports from initial clinical trials involving transplantation of human embryonic stem cell-derived RPE (hESC-RPE) as suspension [7,8] are encouraging and found to be safe for the treatment of AMD and SD. Simple bolus injection of stem/progenitor cell suspension into subretinal space may result in injection reflex and poor cell localization. The compromised cell survival will lead to ineffective cell integration into the damaged retina. Even though cells appear to be well tolerated in relatively short-term animal studies, non-integrated cells will lead to potential complications such as subretinal gliosis [9,10]. To ensure that the cells are in correct orientation and proper interface with the photoreceptor cells, it is desirable to transplant cells as a preformed monolayer along with a supporting substrate. A recent implantation study used, stem cell-derived RPE grown on a bioengineered scaffold (RPE patch), that helps to maintain the polarity and laminar structure of the transplanted RPE cells [9–12]. Results of the on-going Phase1/2a clinical trials indicate good safety and tolerability for surgical implantation of RPE grown on parylene scaffolds [13].

In addition to RPE transplantation, recent advances in the development of pluripotent stem cell (PSC)-derived 3D neural retina in the culture dish and construction of cellular, three-dimensional structures using robotics and 3D bioprinting have provided new insights in the field of tissue engineering of the retina. In this review, we discuss various tissue engineering strategies for retinal repair using stem cell-derived grafts.

## 2. Cell Types Used for Therapies in the Eye

Initial transplantation studies using autologous RPE sheets and RPE isolated from fetal or adult donor eye tissue showed “proof of concept” for photoreceptor preservation and visual functional improvement in human patients [14–21]. Later, transplantation of different stem cell suspensions of the neuronal and non-neuronal lineage including mesenchymal stem cells from umbilical cord [22,23], bone marrow [24–26], adipose tissue [27], human neural progenitor cells [28], embryonic stem cell (ESC)-derived neural progenitors [29], iris pigment epithelium (IPE) derived cells [30,31], and RPE [32,33] were shown to provide trophic support and visual functional improvement in preclinical models of RD diseases. Protocols to differentiate human embryonic stem cells (hESC) and induced pluripotent stem cells (iPSC) to RPE, retinal progenitor cells (RPC), photoreceptor precursor cells, and retinal organoids (RO) have been successfully established by various investigators [34–41].

The RPE cells derived from pluripotent stem cells (PSC) form a monolayer of pigmented cells and show typical features of RPE such as polarity, tight junction formation, and phagocytosis of photoreceptor outer segments [42–44]. Transplantation of pluripotent stem cell-derived retinal cells including RPE, PR, and RO into animal models of retinal degenerative diseases demonstrated their effectiveness in supporting visual function [45–52]. Clinical trials based on subretinal implantation of hESC-RPE and iPSC-RPE as a suspension in AMD and Stargardt’s disease patients showed possible RPE engraftment without significant adverse events [7,53]. There are at least three different clinical trials currently progressing at different centers but they are yet to publish results based on long-term assessments [54–56].

Previous studies have shown that the transplantation of healthy photoreceptor precursors into the diseased retina improves visual function [57–59]. Initial investigators considered this as a result of the donor cell integration into the retina, but later studies proved that donor photoreceptors take part in a cytoplasmic exchange with the host photoreceptors instead of “true integration” [60–62]. Retinal progenitor cells (RPCs) found in the developing neural retina located in the inner layer of the optic cup are capable of differentiating into diverse retinal cell types. Human clinical trials conducted in patients with RP using fetal derived RPCs (fRPCs) demonstrated acceptable safety and tolerability of RPCs [63]. The results of two other clinical trials are on the way (NCT02464436, NCT03073733).

Recent studies show that 3D retinal organoids (ROs) developed from iPSCs and ESCs can produce retinal progenitors that differentiate into RPE, PR, inner nuclear layer (INL) neurons, and ganglion cells (RGCs) [64–67]. In preclinical studies, RO-derived retinal sheets formed structured outer nuclear layers (ONLs) with inner and outer segments [49,51,68]. Transplantation of PRs alone is also appealing but so far only a few protocols to produce PRs are suitable to use in clinical studies [69].

Identifying the right disease stage for cell replacement is an important factor that determines the success of the therapy. In AMD, during the initial stages, only the BM and RPE are affected whereas the photoreceptors (PRs) remain preserved. In this scenario, only RPE replacement may be necessary to cure the disease condition. Combined BM-RPE-PR transplantation may be required for visual recovery in advanced stages of the diseases where the retina is irreversibly damaged (both PR and RPE are dysfunctional).

## 3. Tissue Engineering of the Retina

The environment in which the cells grow and mature can influence their survival and functionality after transplantation. Tissue engineering of the retina is based on the concept that the transplantation of normal healthy cells derived from various stem cell sources needs to be implanted as an intact layer or sheet rather than injected as a suspension. Previously, subretinal delivery of cells through bolus injection has laid the groundwork and provided the “proof of concept” that healthy donor stem and progenitor cells can be transplanted into a diseased retina to contribute to visual functional recovery [70,71]. These preclinical studies emphasized the requirement of improved cell delivery systems to enhance donor cell survival, integration, and neural connectivity.

Advanced AMD is characterized by complete loss of PRs, dysfunctional RPE, and abnormal BM. BM is a 2–4 μm thick extracellular matrix (ECM) composed of collagen types I and IV, laminin, fibronectin, hyaluronic acid, heparan sulfate chondroitin/dermatan sulfate, and elastin [72]. The specialized morphology of BM facilitates the reciprocal exchange of nutrients to and from the retina. In the diseased state, the BM show increased lipid body accumulation and a higher level of collagen cross-linking [73]. The degenerating RPE monolayer and its disrupted tight junctions further alter the BM morphology. These age-related changes result in decreased adhesion and survival of transplanted donor cells [12]. Several groups attempted to resurface BM to facilitate RPE attachment. Although coating the BM with a mixture of laminin, fibronectin, and vitronectin improved cell survival and phagocytosis of fluorescein isothiocyanate (FITC)-labeled bovine photoreceptor outer segments in both adult RPE and fetal RPE, the improvement was not comparable to healthy BM [74].

Transplantation of healthy RPE/PR seeded in a carefully designed scaffold that can mimic the BM morphology and properties can better rescue the deteriorating visual function [9]. The central fovea has a neural retina thickness of 100 μm whereas the BM is only 5 μm [75]. In general, the ideal scaffold should be biocompatible, nonimmunogenic, and mechanically robust enough to resist manipulation during implantation. Scaffolds need to be thin enough to allow the exchange of nutrients and metabolites between the choriocapillaris and the retina [76]. After transplantation, it should not lead to physical distortion of the photoreceptor layer. Low elasticity of the material prevents adverse events like retinal detachment, retraction, or visual distortion. Carefully designed, cutting-edge biomaterials with fine-tuned topographical properties and micro/nanopatterned structures with extracellular matrix (ECM) properties can hold stem and progenitor cell populations effectively and help to deliver them as a retinal patch into the subretinal space.

Different types of biomaterials have been used to design scaffolds for retinal tissue engineering. This includes natural polymers, synthetic polymers, hybrid polymers, decellularized tissues, and thermoresponsive hydrogel polymers.

## 4. Biomaterials and Scaffolds Used for Tissue Engineering

### 4.1. Natural Biomaterials Used as Scaffolds

Biomaterials mainly include ECM proteins and polysaccharides which possess bioactive properties. The natural polymers used for retinal tissue engineering are easily available and include collagen types I, III, and IV, gelatin, alginates, laminin, fibronectin, matrigel, silk fibroin, and vitronectin. These scaffolds constitute nanofibers that have very similar physiological properties as BM in terms of morphology, mechanical properties, protein concentrations, and biocompatibility. Collagen I is a major component of the inner collagenous layer of BM and studies have proved them as a viable substrate for RPE cell reattachment [77]. Usually, this polymer is too thick for subretinal implantation, hence specially designed ultrathin (7 μm) membranes were designed for testing. Thumann et al. [78] showed that ultrathin collagen membranes can remain stable for at least 10 weeks and completely degrade within 24 weeks. By then the transplanted RPE were able to restructure the BM. In another study, collagen films supported by Teflon showed RPE attachment and viability [79]. Human primary RPE cells and the immortalized retinal pigment epithelial cell line (ARPE-19) have been previously cultured on equine, bovine, and rat collagen type I membranes as well as on human collagen type I thin films [80,81]. Gelatin, a denatured form of collagen proteins, is advantageous over collagen because of lower immunogenicity, crosslinking ability, and better solubility in aqueous systems. Gelatin membranes in the shape of a sandwich with encapsulated retinal grafts were used for transplantation studies in rabbits to demonstrate biocompatibility, improved survival, and formation of laminar structures [82]. Gelatin membrane cross-linked with carbodiimide when used for retinal sheet implantation was found to be more stable against hydrolysis and mechanical stress [83].

Alginate is an anionic polysaccharide that is usually found in the cell walls of brown algae. In a study, a thin film of purified alginate was used to demonstrate its ability to support the growth of RPE cells and their high proliferative rates [84]. In another study, alginate beads were used to demonstrate RPE cell sustenance and proliferation [85]. An arginine-glycine-aspartic-alginate (RGD-alginate) scaffold demonstrated feasibility for cell derivation and transplantation of RPE and neural retina [86]. Bombyx mori silk fibroin (BMSF) that possesses unique structural properties and mechanical strength, is another suitable candidate to be used in the eye [87]. BMSF pre-coated with vitronectin is used to fabricate a membrane up to 3 μm in thickness as a carrier substrate for human RPE transplantation. Although the cells were grown on BMSF for approximately 8 weeks with expressing RPE characteristics, the duration required to establish the culture was comparatively long [88].

Recent advances in decellularized scaffold techniques are expected to better preserve tissue architecture and chemistry. Kundu et al. [89] used ionic detergents to decellularize bovine eyes and processed them into stable thin films. The decellularized matrix-supported adherence and proliferation of human RPCs. The gene expression of CRX, ROM1, RHODOPSIN, and NRL on these retinal films indicated photoreceptor differentiation [90]. In another study, an amniotic membrane was used as a BM substitute, in which it supported RPE ingrowth in the pig eyes with choroidal neovascularization [90]. Areas of hypo and hyperpigmentation observed in this study were attributed to the migration of RPE cells into the affected region in the presence of an amniotic membrane. Interestingly, the amniotic membrane was not stated as being beneficial or detrimental to choroidal neovascularization, as there was an initial hemorrhage but no additional leakage [90].

### 4.2. Synthetic Biomaterials Used as Scaffolds

Synthetic scaffolds have better mechanical properties that can resist the transplantation procedure. Suitable bulk properties can be obtained in a controlled way by modifying the porosity, topographical parameters, and dimensional shape. Synthetic scaffolds are more advantageous than natural scaffolds because of their reproducibility and longer shelf life. Biocompatible, inert materials can be free from immunogenicity and their biodegradation rates can be manipulated. A number of polymers meet many of these requirements and have been approved by the food and drug administration (FDA) for an array of biodegradable suture applications including poly(lactic-co-glycolide acid) (PLGA), poly(l-lactic acid) (PLLA), PLLA–PLGA copolymer systems, poly(glycerol-sebacate) (PGS), polydimethylsiloxane (PDMS), polydimethylsiloxane (PDMS), poly(methyl methacrylate) (PMMA), poly(ethylene glycol) diacrylate (PEGDA), parylene-C and polycaprolactone (PCL).

PLGA is a biodegradable polyester-based polymer having remarkable mechanical properties, adjustable degradation rates, and good processability [91]. It degrades by hydrolysis of ester linkages forming lactic and glycolic acids which are further degraded in the body. By varying the amount of lactic and glycolic acids, the degradation rate can be controlled. A clinical-grade PLGA scaffold was seeded with AMD patient–derived iPSC-RPE to demonstrate safety and cell integration in the eye [45]. This cell patch showed improved efficacy in rodent and porcine preclinical models. Concurrent PLGA scaffold degradation and ECM production by the donor cells aided integration with the host BM [45]. Biodegradable PCL scaffolds are the thinnest scaffolds available for retinal tissue engineering. This will act as a permeable and slowly degrading transient structure without any pathological increase in local acidity [92]. Bernards et al. [93] conducted *in vivo* studies in rabbits to assess the tolerance and durability of micro and nanostructured PCL thin films. Adverse tissue responses like fibrosis or biodeposits were not observed and a good ocular tolerance was observed.

Poly (trimethylene carbonate) (PTMC) is flexible and elastic in nature and a biodegradable polymer. In one of the studies, PTMC film was compared with an often-used biodegradable polymer namely poly (D, L-lactide) (PDLLA) film. The mechanical properties of PTMC film were found to be comparable to that of native Bruch’s membrane (BM) and it also supported the formation of a functionally active hESC-RPE monolayer. On the other hand, PDLLA did not support the formation of hESC-RPE merging monolayers and had inappropriate mechanical properties when used for in vivo applications [94].

Synthetic polymers are generally hydrophobic in nature and usually not favorable for cell attachments. Oxygen-plasma processing, hydrogel blending and surface modifications such as coating surface with extracellular matrix proteins allow greater cell adhesion and survival. Tao et al. [95] designed thin PMMA scaffolds of 6 μm that were easy to implant and with reduced risk of trauma after transplantation into the rodent eyes. RPC on laminin-coated porous scaffolds resulted in increased cell survival and the delivery could be localized to specific retinal regions. Redenti et al. [96] generated a laminin-coated novel biodegradable nanowire PCL scaffold on which mouse RPCs were cultured. A microfabricated, elastic poly (glycerol sebacate) (PGS) scaffold was found suitable for initial RPC differentiation in vitro. Subretinal delivery into C57bl/6 and rhodopsin knockout mice allowed the passage of nutrients and cells through its 50 μm diameter pores. Polymer topology allowed photoreceptor maturation and migration of RPC into the retina demonstrating localized delivery of a predetermined number of cells to a specific region of the damaged retina. PGS has proved to be a potential scaffold for RPC delivery as indicated by high levels of survival, adherence, and proliferation [97]. Lavik et al. [98] showed that RPCs seeded on PLLA-PLGA copolymers, down-regulated immature “stemness” cell markers (Hes5, nestin, Hes1, and Pax6), and upregulated mature retinal markers such as glial fibrillary acidic protein (GFAP), nevertheless, there was no photoreceptor-specific expression [98].

**Figure 1.**
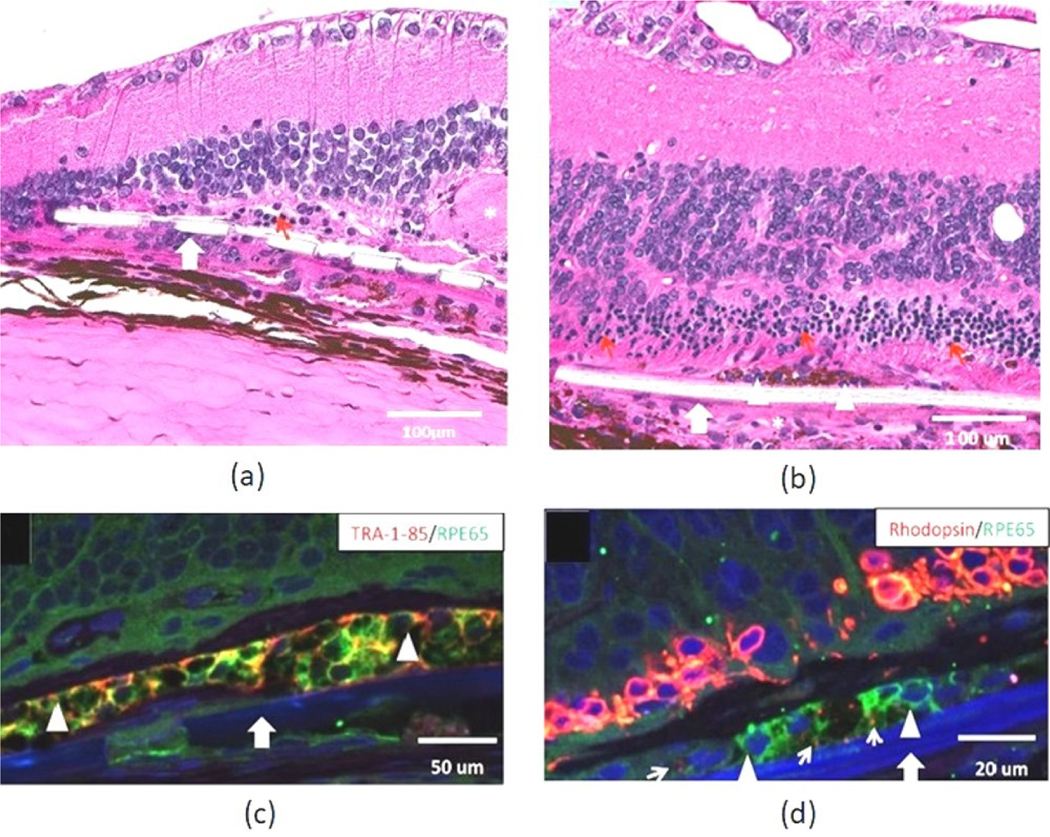
Histologic assessment of Mesh-supported submicron parylene C membranes (rMSPM)+ Vitronectin and California Project to Cure Blindness–Retinal Pigment Epithelium 1(rCPCB-RPE1) implants in Royal College of Surgeons (RCS) rats. Representative hematoxylin eosin (HE) staining images of rat retina after implantation. Implanted (**a**) parylene membrane (rMSPM+ Vitronectin) and (**b**) rCPCB-RPE1 in the subretinal space (large white arrow), surviving outer nuclear layer (ONL) (red arrows), and an area showing some cellular reaction (white stars). Relatively intact host retina, elevated and wavy inner nuclear layer (INL) and focal loss of INL cells can be observed in both (**a**,**b**). The choroidal layer that appears to be separated from the remaining retina is considered a histologic artifact. (**c**) Immunostaining of TRA-1–85/RPE65 shows implanted hESC-RPE cells (white arrowhead). (**d**) Rhodopsin immunostaining showing rhodopsin-positive phagosomes inside the implanted RPE65-positive hESC-RPE cells (small white arrow pointing to phagosomes) (reprinted with permission from Thomas et al., 2016).

Parylene-C is a class VI biocompatible polymer having several biomedical applications including the fabrication of Argus^®^ II Retinal Prosthesis System [99]. Our preclinical and translational studies showed that composite implant of RPE and parylene is a feasible option to rescue visual function [9,13,100,101]. The parylene material used was semipermeable to molecules of a certain molecular weight when its thickness was reduced to a sub-micron range. Lu et al. [102] designed a mesh-supported sub-micron parylene-C membrane (MSPM). To get better cell adherence, both sides of the membrane were treated with low-power oxygen plasma and coated with matrigel. RPE cells demonstrated good adherence and showed epithelial-like morphology. They developed microvilli, right polarization, and tight intracellular junctions [102]. Survival of the transplanted RPE cells in the subretinal space of Royal College of Surgeons (RCS) rats up to 21 weeks post-implantation was demonstrated by Thomas et al. [100]. The implanted hESC-RPE cells remained as a monolayer on the surface of the parylene substrate and performed photoreceptor outer segment phagocytosis (Figure 1). A partially blinded randomized study was conducted on Yucatan minipigs before human clinical trials. Results showed structural preservation of the implant; the RPE cells remained intact and survived in the form of a monolayer [103]. Following this, an RPE+ parylene implant named California Project to Cure Blindness–Retinal Pigment Epithelium 1 (CPCB-RPE1) was developed for use in clinical studies which are currently being conducted in patients with dry AMD.

In another in vitro study, specifically designed porous honeycomb PLA films coated with collagen IV were seeded with pigmented hESC, showed cell survival and proliferation during the 6 weeks of the study period [104]. A biomimetic scaffold sheet of plasma modified polydimethylsiloxane (PDMS) coated with laminin was found to facilitate the functional maturation and survival of RPE cells [105]. PCL–gelatin scaffold, poly(lactic-co-glycolic acid) (PLGA)-collagen type I, poly(ethylene glycol) diacrylate (PEGDA)- RGDS peptide motif (arginine-glycine-aspartic acid-serine) are the other polymer types shown to support RPE survival and maturation [106–108].

Human retinal progenitor cells (hRPCs), isolated from the fetal retina, need extracellular matrix proteins such as fibronectin or laminin for attachment and survival. A synthetic, xeno-free vitronectin-mimicking surface (Synthemax) was fabricated by Baranov et al. to grow RPCs; RPCs survived and self-renewed in the in vitro condition [109]. In another study, the authors used xeno-free synthetic RGD peptides to coat the PCL scaffold. The coating promoted the differentiation of rods in vitro but not the differentiation of cones or other retinal cell types. The expression of stem cell markers KLF4 and N-MYC remained high due to which this construct is considered undesirable for human applications [110].

### 4.3. Biohybrid Scaffolds

Hybrid scaffolds have the advantage of combining the properties of both natural and synthetic nanofibers by incorporating both materials to make composite scaffolds. This combination approach is different from coating the synthetic scaffolds with natural materials like extracellular matrices or proteins. The combination approach allows tailoring scaffold properties of the synthetic component and gaining natural properties of proteoglycans, proteins, and glycosaminoglycans from the natural polymer. Thomson et al. [111] manufactured five blends of PLLA with PLGA to evaluate a variety of suitable scaffolds for RPE transplantation. The blend with a 25:75 (PLLA:PLGA) ratio was found to be the thinnest and most porous with minimal cell death [111].

A novel scaffold fabricated from Silk Fibroin (SF) and Poly(L-lactic acid-co-ε-caprolactone) (PLCL, 1:1) showed RPC growth, proliferation, and differentiation into photoreceptors [112]. A cationic chitosan-graft-poly(ɛ-caprolactone)/polycaprolactone (CS-PCL/PCL,20/80) hybrid scaffold produced using electrospinning fabrication technique demonstrated great RPC proliferation [113]. Previous studies have demonstrated that PCL with laminin and PCL with chitosan electrospun nanofibers, can improve cell adhesion, proliferation, or differentiation and promote the expression of genes specific to photoreceptor cells or bipolar cells [96,113,114]. Issues related to reproducibility and batch variability while using natural polymers exist in the combination approach. Future studies should address these issues along with effective measures to control the biodegradability and immunogenicity of the by-products of combination scaffolds. A summary of different biomaterials used for retinal tissue engineering is included in Table 1.

### 4.4. Scaffold Free Cell Sheets Using Thermoresponsive Polymers

**Figure 2.**
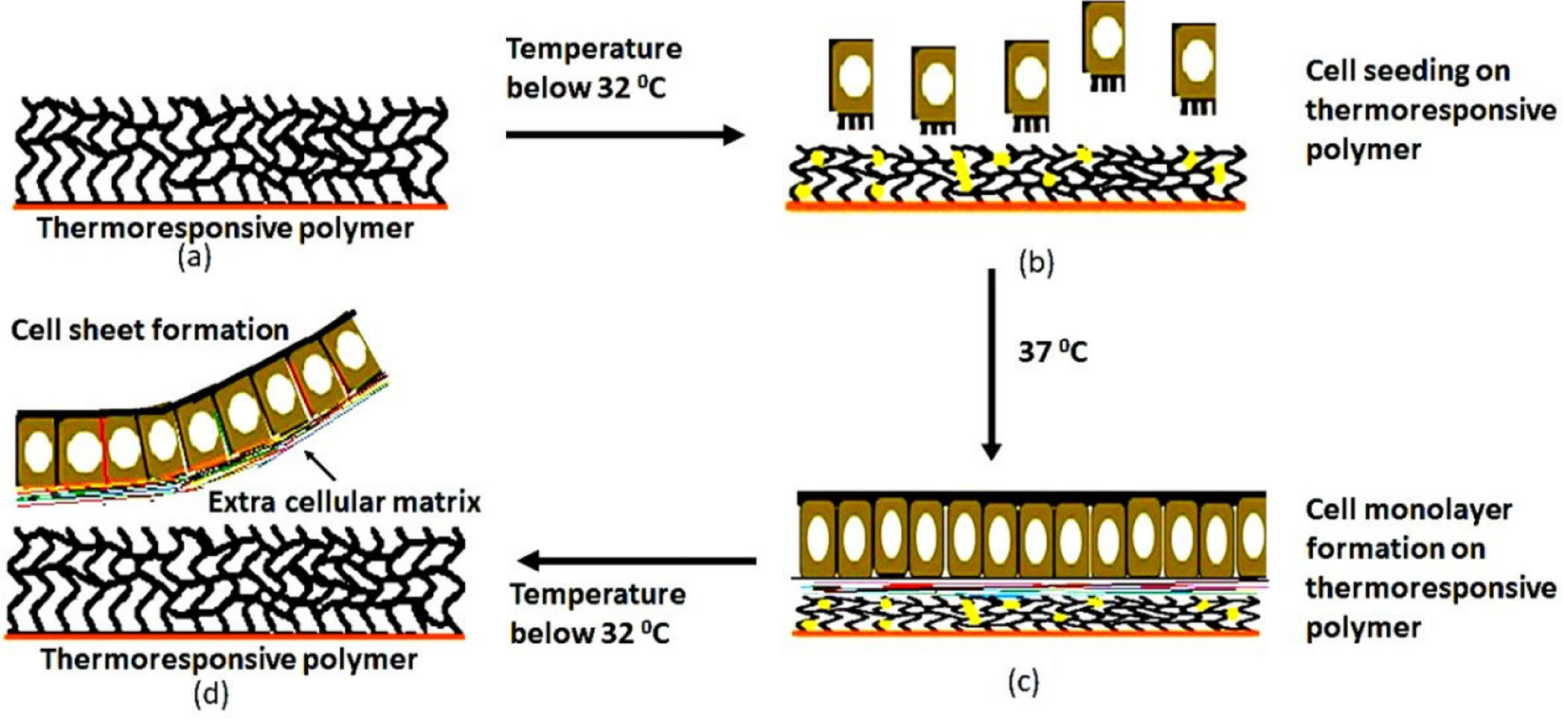
Diagram illustrating the concept of temperature-dependent cell-sheet detachment. (**a**) Preparation of thermoresponsive polymer; (**b**) Cell seeding into the polymer at a temperature below 32 °C (**c**) Schematic diagrams for the interactions of the thermoresponsive surface with the cells growing on it (**d**) Cell sheet detachment from the thermoresponsive cell culture dish, where the cell sheet retains the extracellular matrix and cell–cell junctions.

Thermoresponsive polymers are stimuli-responsive smart materials that show reversible hydrophilicity/hydrophobicity around a critical temperature [115] (Figure 2). This can be used to prepare cell monolayers or sheets for implantation without any supporting matrix. The approach can enable the preparation of intact, scaffold-free monolayer cell sheets along with the deposited ECM through phase separation. During transplantation, the ECM supports faster attachment of host tissue without any additional coating. Poly(*N*-isoproplyacrylamide) (PNIPAAm) is one of the most popular thermoresponsive polymers which allows cell sheet harvest by temperature reduction from 37–20 °C [116]. Kubuta et al. [117] has shown that RPE forms cell sheets over PNIPAAm and exists as a monolayer structure with intact cell-to-cell junctions after transplantation [117]. Functional three-dimensional (3D) tissues can also be fabricated using thermoresponsive polymers by layering cell sheets. Micro-patterning technology combined with cell sheet technology can be used to create more complex 3D functional tissues [118].

### 4.5. Co-Graft of RPE and Retinal Organoid

**Figure 3.**
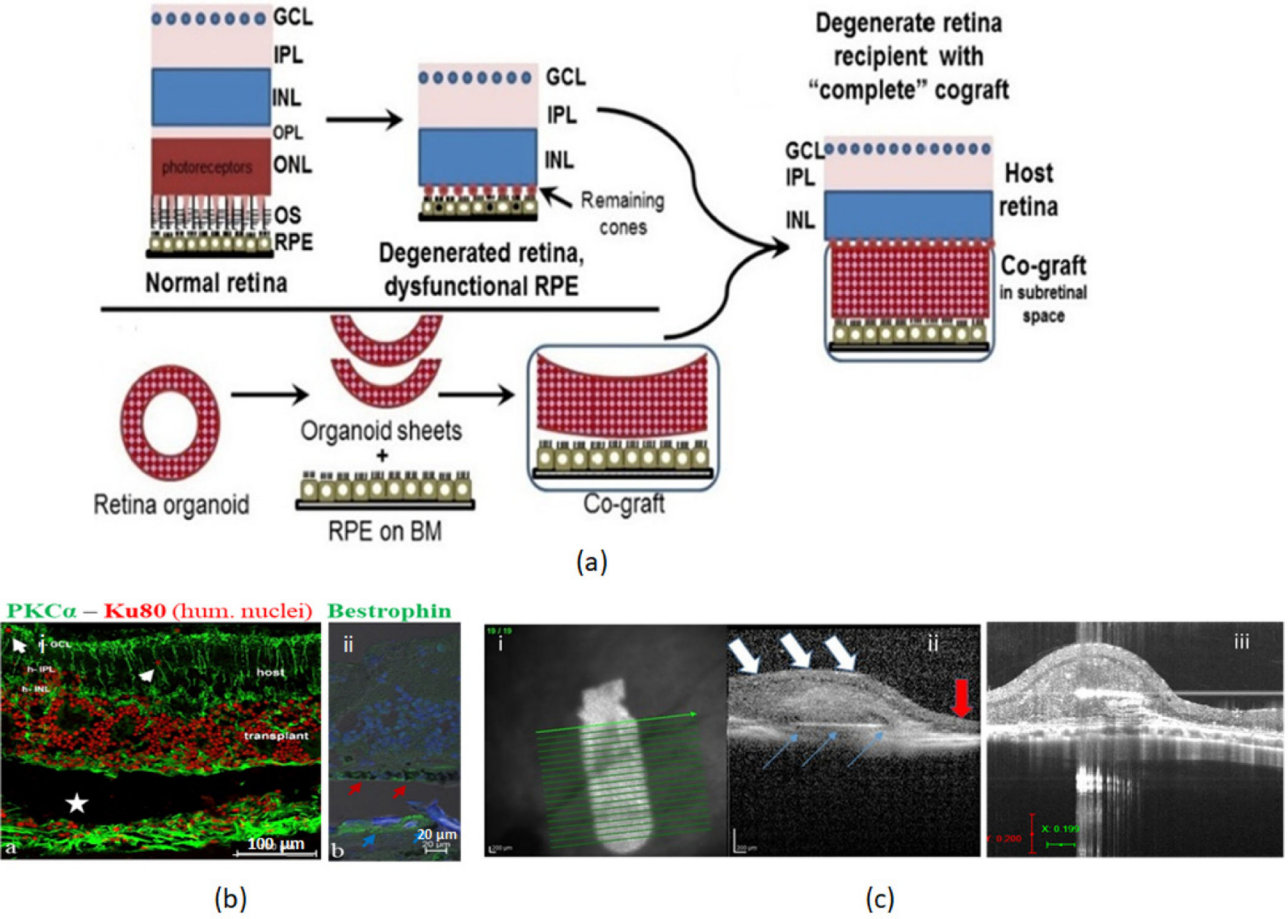
Tissue- engineered RPE-Retinal organoid co-graft transplantation into subretinal space of RCS rats (**a**) schematic representation, showing the transplantation of RPE-RO cograft into the degenerated retina. After transplantation to the subretinal space, both pieces integrate to repair a damaged retina. GCL: ganglion cell layer, IPL: inner plexiform, INL: inner nuclear, OPL: outer plexiform, ONL: outer nuclear, OS: outer segments, RPE: retinal pigment epithelium, BM: basement membrane. (**b**) Immunohistochemical staining showing RPE-RO co-graft integration into the degenerative rat retina, 3 months post-implantation. (**i**) in (**b**) co-graft (red nuclei) in subretinal space of rat. The transplant has developed rosettes. White arrows: migrated donor cells in the host (**ii**) in (**b**) bestrophin (green) shows donor RPE (red arrows) and host RPE (blue arrows). (**c**) Ultrasound images after ROE-RO cograft transplantation. (**i**) in (**c**) ultrasound image of a co-graft observed during fundus examination of RCS rat- 3 months post-implantation) (**ii**) in (**c**) vertical OCT B-scan image passing through the transplant area. Blue arrow: RPE layer on synthetic Bruch’s membrane, white arrow: organoid layer above RPE. Here the RPE-RO co-graft area appears like a normal retina whereas the outside area (indicated by the red arrow) shows considerable loss of retinal thickness. (**iii**) in (**c**) Vertical OCT B-scan image of another RPE-RO cograft transplant at 5 months post-surgery (unpublished data).

During advanced stages of AMD, when both PR and RPE are lost, RPE replacement alone may not rescue vision. Retinal repair at this stage requires transplantation of both tissues. Designing better models of photoreceptor-RPE interaction for transplantation is an important goal that needs an urgent solution, for the treatment of advanced geographic atrophy. Retinal organoids (ROs) are a considerable source of photoreceptor precursor cells, but they lack a continuous and mature layer of RPE [119]. Using a co-graft made of RO sheet and RPE is beneficial since it can address both the lack of photoreceptors and RPE. In our lab, a composite graft made of RO sheets and polarized RPE sheets cultured over parylene is used as a composite implant to determine its potential to repair retina and rescue vision(unpublished data) in preclinical animal models of retinal degeneration (Figure 3).

## 5. Other Complex Tissue Engineering Approaches

Drop casting [120], solvent casting [120], electrospinning [113], soft lithography [120], and microfabrication [97] are some of the techniques conventionally used to produce scaffolds for retinal repair. These methods can be used to fabricate porous scaffolds. The pore size and porosity can be controlled by choosing the correct particle size and the right number of added particles. The robotic deposition is an upcoming technology in tissue engineering for computerized and reproducible patterning of ultrathin membranes for cell delivery [115]. This will allow controlled cellular deposition in micrometer levels. Cell adhering surfaces can be manipulated to tailor the alignment and morphology of the attached cells through the introduction of cell-aligning grooves. The viable cells are delivered through a bio-ink which consists of a biocompatible polymer [121]. In 3D bioprinting, the components are fabricated by layers directly from a computer-assisted design file [120]. 3D bioprinting allows combining cells, biomaterials, and growth factors to mimic the natural tissue characteristics. Conventional methods lack precision and the ability to create constructs having complex designs. Since the structure of the retina is complex with a heterogeneous cell population and degenerative diseases affecting photoreceptors, RPE, choriocapillaris, and BM; 3D bioprinting technology can be applied to repair the damaged retinal layers. Using 3D bioprinting, Shi et al. printed a retina model composed of a PCL ultrathin membrane, Y79 cell-laden alginate/pluronic bio-ink, and ARPE-19 cell monolayer with potential applications in drug delivery, disease mechanism, and treatment method discoveries [122]. In another study, to develop an in vitro retina model, an inkjet bioprinting system was applied to PR cell layers placed on top of bioprinted RPE. Results showed well-positioned layered structures expressing their structural markers. Human vascular endothelial growth factors were released from RPE printed layer confirming a functional RPE monolayer obtained by bioprinting [123].

## 6. Current Clinical Trials Using Biomaterial Scaffolds

In a Phase1/2a clinical study, Kashani et al. [13] implanted clinical-grade hESC-RPE grown on 3.5 mm × 6.25 mm parylene membrane substrate (CPCB-RPE1) in five patients suffering from geographic atrophy (GA) associated with advanced non-neovascular AMD. Postoperative findings demonstrated that there is no progression of vision loss. In one eye, the improvement was seen by 17 letters, and improved fixation was seen in two eyes. The appearance, size, position of the implant also did not change, and no adverse events were noted [13]. Da Cruz et al. and his team engineered a 6 mm × 3 mm RPE patch which constitutes of differentiated hESC derived RPE monolayer placed on a human-vitronectin-coated polyester membrane (polyethylene terephthalate, PET). The patch delivered to the subretinal space of the retina in patients with AMD using a microsurgical tool survived and integrated with the host retina. There was a focal improvement in photoreceptor anatomy over the transplant in both patients with a visual acuity improvement of 15 letters or more [124].

In another clinical trial study from RIKEN Center for Developmental Biology (Japan), iPSC-RPE cells were prepared as a sheet by growing them on collagen support. After confluence, the cells were treated with collagenase to obtain a cell sheet on its own ECM. The cell sheet was transplanted along with immunosuppression in a patient suffering from neovascular AMD. When it was assessed at one year, the sheet remained intact, but the best-corrected visual acuity had neither improved nor worsened. However, the trial was forced to stop later because of mutations noticed in the second patient’s iPSCs and due to changes in the regulatory rules in Japan [53]. In all the above clinical trials, specialized surgical tools and devices were used for implant delivery. These devices minimized the extent of the retinotomy and allowed precise positioning [125,126]. The main endpoints of these studies were safety and some efficacy. In future studies, large multicentral clinical trials with more patients are needed to measure the efficacy and statistical significance of advanced phase clinical trials.

## 7. Challenges and Future Directions

Transplanted cells in the retina perform better in terms of physiology and cell survival when they are supported by a scaffold, compared to cell suspension. Support from factors provided by a cell monolayer (such as extracellular matrix and adhesion molecules) can help the cells to function better when transplanted along with the substrate. There are different methods to construct a scaffold including spun, machined, printed, assembled stepwise, or casted. New methods to create microscale niches for cocultured stem cells are also being explored. In the future, robotic and 3D bioprinting will allow several multiple types of cells and tissue layers to be combined with new generation scaffolds, to construct complex implants. Many scaffolds discussed here have not been assessed in vivo and therefore, evaluation of each type of scaffold is required in animal models to establish “the proof of concept”. Implanting polymer scaffolds thicker than the size of the retina may result in retinal trauma and detachment during surgery or during the post-surgery period. It is also important to rule out inflammation caused due to scaffolds and their by-products. Using fast degrading polymers for clinical applications is limited mostly due to toxicity issues [127]. Even though many fast degrading polymers are in the development stage, slow degrading polymers might show lesser adverse events after transplantation. PLGA and PGA have faster rates of degradation compared to PCL or PLA polymers (over 2 years).

Natural polymers have limited processability and it is difficult to control their batch to batch variability and mechanical properties. There can be changes in the constituents of natural polymers with age which can cause accumulation of debris in BM leading to the dysfunction of the transplanted cells [73]. The poor mechanical properties of most of the natural polymers make them difficult to handle surgical procedures. Methods like cross-linking are used to improve mechanical properties but this can make them thicker, poorly permeable, and non-biodegradable. Transplantation of tissue-engineered scaffolds into the retina needs immunosuppressants at least provisionally until the blood-retinal barrier heals [128]. In the future, different HLA-matched, genetically screened, cGMP grade PSC-derived cells from the initial passages can be made available in cell banks, which will make cross-matching easy, to find the most suitable cells to avoid an immune response. Bringing together appropriately layered RPE with the multi-layered neural retina and establishing connections with the retinal ganglion cells for the visual signals to reach the brain through the optic nerve are the major challenges in tissue engineering the retina. Fine-tuning of the combined aspects of advancements in material science, stem cell biology, and clinical expertise along with the inputs from the ongoing clinical trials can resolve the hurdles in developing a final clinical-grade protocol for the therapy of retinal degenerative disease.

## 8. Conclusions

Retina tissue engineering is expected to make significant contributions to the treatment of human blindness, especially for RD diseases in which RPE and/or PRs need to be replaced. Most of the current clinical trials are in the early I/IIa phases. There is still a long way to go before these findings can be applied to clinical practice. As the confirmed biosafety and feasibility of RPE and RPC transplantation has laid a solid foundation for vision repair, the next step is to enhance the visual improvements observed in RD patients. There are still restrains regarding the appropriate cell type and method to be used to improve neural integration with the host retina. Establishing robust and reproducible protocols for the production of cGMP-grade hPSCs derived RPE/organoids from stem cell banks, with normal karyotype without genetic abnormalities is a primary requirement. In the future, the concept of making combinations of RPE/PR/BM microscale niches using 3D bioprinting can be a suitable approach to bring functional (synaptic) integration with the host neural circuitries leading to improved visual function.

## Figures and Tables

**Table 1.** A summary of biomaterials used for retinal tissue engineering. PLGA: poly(lactic-co-glycolide acid), PLLA: poly(l-lactic acid), PGS: poly(glycerol-sebacate), PTMC: Poly (trimethylene carbonate), PCL: polycaprolactone, PMMA: poly(methyl methacrylate), SF: silk fibroin, PDLJA: poly (D, L-lactide), PLCL: poly(L-lactic acid-co-ε-caprolactone, hESC: human embryonic stem cell, RPE: retinal pigment epithelium, RCS: royal college of surgeons, BM: Bruch’s membrane, BMSF: bombyx mori silk fibroin, RPC: retinal progenitor cells, AMD: advanced macular degeneration, PNIPAAm: poly(*N*-isoproplyacrylamide).

Biomaterial	Thickness (μm)	Advantages	Studies	References

Collagen type I membrane	7	Non-toxic, no inflammatory response, controllable, stability (10 weeks), degrade (within 24 weeks)	Long term biocompatibility and membrane degradation evaluated (rabbits)	(Bhatt et al., 1994; Booij et al., 2010; Lu et al., 2007; Thumann et al., 2009)

Gelatin	30–35	Lower immunogenicity, crosslinking ability, and better solubility in aqueous systems	Biocompatibility, improved survival, and formation of laminar structures (rabbits)	(Hsiue et al., 2002; Lai and Li, 2010)

Alginate	Thin film	Purified alginate- high cell proliferative rate	Ability to support the growth of RPE cells and their high proliferative rates (in vitro)	(Heidari et al., 2015; Hunt et al., 2017; Jeong et al., 2011)

Silk Fibroin	3	Great mechanical strength, good biodegradability, and biocompatibility	Evaluate BMSF as a substrate for RPE cell transplantation (in vitro)	(Shadforth et al., 2012; Tran et al., 2018)

PLGA		Remarkable mechanical properties, adjustable degradation rates (80–90 days), and good processability	To demonstrate safety and cell integration in the eye (rodent and porcine preclinical models)	(Pan and Ding, 2012; Sharma et al., 2019)

PCL	20–40	Thinnest scaffold, permeable, slow degradation, adverse tissue responses not observed	Assess the tolerance and durability of micro and nanostructured PCL thin films (rabbits)	(Bernards et al., 2013; Redenti et al., 2008)

PTMC	100	Elastomeric properties similar to BM, thickness tunable	Demonstrate adherence and maturation of hESC-RPE cells on PTMC compared to PDLLA films	(Sorkio et al., 2017)

PMMA	6	Reduced risk of trauma	Evaluate adhesion of RPCs and its differentiation and migration to host retina (mice)	(Tao et al., 2007)

PGS	45	A suitable candidate for RPC delivery with great novel properties	Evaluate mechanical properties	(Neeley et al., 2008)

Parylene-C	0.15–0.30, 0.3 μm thickness supported on a 6.0 μm thick mesh frame	Macromolecules and nutrients can diffuse, nonimmunogenic, Promotes cell adhesion after vitronectin/matrigel coating	Evaluate safety, survival, and functionality of hESC-RPE cells on parylene in animal models	(Kashani et al., 2018; Koss et al., 2016; Lu et al., 2012; Thomas et al., 2016)
	
	0.3 μm thickness supported on a 6.0 μm thick mesh frame		Assess safety and efficacy of hESC-RPE on parylene in patients with AMD. (clinical study)	
	
			Check cell adherence and proliferation (in vitro)	

PLLA & PLGA	Week 1: 133.1 Week 2: 131.5 Week 3: 103.5	25:75 (PLLA: PLGA) thinnest, most porous, and minimal cell death	Evaluate the variety of suitable scaffolds for RPE transplantation (in vitro)	(Thomson et al., 2011)

SF & PLCL	60–100	Quick RPC proliferation, preferential differentiation towards retinal neurons like photoreceptors	Understand effects of blended nanofibrous membranes of silk fibroin and PLCL (in vitro)	(Zhang et al., 2015)

Honeycomb like films and collagen IV		Increased hydrophilicity, high permeability	Investigate honeycomb-like film as a promising scaffold for hESC-RPE tissue engineering	(Calejo et al., 2016)

PNIPAAm - Thermoresponsive polymer	scalable	Allows cell sheet harvest by temperature reduction from 37–20 °C	Demonstrate fabrication of transplantable retinal pigment epithelium cell sheets	(Kubota et al., 2006; Kushida et al., 1999)

Decellularized matrix	10–20	micro- and macro-scale structural components and functional ECM proteins present Photoreceptor differentiation	Develop novel biomaterial by decellularizing retina using ionic detergents	(Kundu et al., 2016)
